# Association between lipoprotein(a) (Lp(a)) levels and Lp(a) genetic variants with coronary artery calcification

**DOI:** 10.1186/s12881-020-01003-3

**Published:** 2020-03-27

**Authors:** Sonali Pechlivanis, Amir A. Mahabadi, Per Hoffmann, Markus M. Nöthen, Martina Broecker-Preuss, Raimund Erbel, Susanne Moebus, Andreas Stang, Karl-Heinz Jöckel

**Affiliations:** 1 Institute for Medical Informatics, Biometry and Epidemiology, University Hospital of Essen, University Duisburg-Essen, Essen, Germany; 2 Institute of Pharmacology and Toxicology, Centre for Biomedical Education and Research, Witten/Herdecke University, Witten, Germany; 3 Department of Cardiology and Vascular Medicine, West German Heart and Vascular Center, University Hospital of Essen, University Duisburg-Essen, Essen, Germany; 4 Department of Genomics, Life & Brain Center, University of Bonn, Bonn, Germany; 5 Division of Medical Genetics, Department of Biomedicine, University of Basel, Basel, Switzerland; 6 Department of Clinical Chemistry and Laboratory Medicine, University Duisburg-Essen, Essen, Germany; 7 Centre for Urban Epidemiology, University Hospital Essen, Essen, Germany; 8 Centre for Clinic Epidemiology, University Hospital of Essen, Essen, Germany

**Keywords:** Lp(a), *LPA* genetic variants, Coronary artery calcification

## Abstract

**Background:**

To examine the association between lipoprotein(a) (Lp(a)) levels, *LPA* (rs10455872 and rs3798220) and *IL1F9* (rs13415097) single nucleotide polymorphisms (SNPs) with coronary artery calcification (CAC), an important predictor for coronary artery disease (CAD).

**Methods:**

We used data from 3799 (mean age ± SD: 59.0 ± 7.7 years, 47.1% men) Heinz Nixdorf Recall study participants. We applied linear regression models to explore the relation between the log-transformed Lp(a) levels and *LPA* and *IL1F9* SNPs with log_e_ (CAC + 1). The association between the SNPs and log-transformed Lp(a) levels was further assessed using linear regression. The models were adjusted for age and sex (Model 1) and additionally for Lp(a) levels (Model 2).

**Results:**

We observed a statistically significant association between log-transformed Lp(a) levels and CAC (Model 1: beta per log-unit increase in Lp(a) levels = 0.11; 95% confidence interval [95% CI] [0.04; 0.18], *p =* 0.002). Furthermore, the *LPA* SNP rs10455872 showed a statistically significant association with CAC (Model 1: beta per allele = 0.37 [0.14; 0.61], *p =* 0.002). The association between rs10455872 and CAC was attenuated after adjustment for Lp(a) levels (Model 2: beta per allele = 0.26 [− 0.01; 0.53], *p =* 0.06). Both *LPA* SNPs also showed a statistically significant association with Lp(a) levels (Model 1: beta_rs10455872_ per allele: 1.56 [1.46; 1.65], *p <* 0.0001 and beta_rs3798220_ per allele: 1.51 [1.33; 1.69], *p <* 0.0001)). The Mendelian randomization analysis showed that Lp(a) is a causal risk factor for CAC (estimate per log-unit increase in Lp(a) levels (95% CI), *p*: 0.27 [0.11; 0.44], *p =* 0.001). The *IL1F9* SNP did not show any statistically significant association with Lp(a) levels or with CAC.

**Conclusions:**

We provide evidence for the association of *LPA* rs10455872 with higher levels of Lp(a) and CAC in our study. The results of our study suggest that rs10455872, mediated by Lp(a) levels, might play a role in promoting the development of atherosclerosis leading to cardiovascular disease events.

## Background

Lipoprotein(a) (Lp(a)) is a complex particle and has similarities with apolipoprotein (apo) (a) and apoB linked by a disulfide bond [1]. The role of Lp(a) is well established for the risk of coronary artery disease (CAD) [2, 3]. Genome-wide association studies (GWAS) have identified two single nucleotide polymorphisms (SNPs) at the Lp(a) locus (*LPA*) on chromosome 6q26–27 (rs3798220 and rs10455872) that were strongly and independently related to Lp(a) levels and with the risk of CAD [3–7]. Coronary artery calcification (CAC) is an important predictor of CAD, and its extent is directly related to the atherosclerotic plaque burden. Quantification of CAC has been shown to allow better risk prediction of future cardiovascular disease (CVD) events [8, 9]. Furthermore, studies have examined the association of Lp(a) with CAC and have shown conflicting results [10–14]. Kullo et al. and Guerra et al. showed no relationship between Lp(a) and CAC score [13, 14]. However, Erbel et al., Greif et al. and Alonso et al. showed a positive relationship between Lp(a) and CAC score [10–12]. In a GWAS, the *LPA* rs10455872 SNP was associated with aortic valve calcification (AVC) [15]. In this study, two other SNPs that are in high linkage disequilibrium (LD) near the proinflammatory gene (*IL1F9)* (rs17659543 and rs13415097) also achieved GWA significance with mitral annular calcification (MVC). Both AVC and MVC have been associated with the risk of CVD [16–18].

Understanding the effects of Lp(a) as well as the SNPs in the LPA and IL1F9 genes on CAC might provide insight into the mechanisms by which they cause CAD. Hence, the aim of our study was to examine the association between the *LPA* and *IL1F9* SNPs with CAC in relation to the Lp(a) levels by using the data of the population-based Heinz Nixdorf Recall study participants.

## Methods

### Study population

As described previously, at baseline examination which was carried out between December 2000 and August 2003, 4814 participants aged between 45 and 75 years (50% women) were randomly selected from the registration lists of the densely populated Ruhr metropolitan cities (residents of Essen, Bochum, and Mülheim an der Ruhr) in Germany. The rationale and design of the study were previously described in detail [19, 20]. For this study, we included data from the Heinz Nixdorf Recall Study participants with Lp(a) and CAC measured at baseline. We further excluded participants having prior CAD (coronary artery bypass surgery and/or interventional revascularization, history of prior myocardial infarction or stroke) (*n* = 432) at baseline. Due to the apparent discrepancy between the evidence in the observational and genetic studies regarding the link between *LPA* and plasma levels of Lp(a) with the risk of ischemic stroke we further excluded incident stroke (*n* = 131) from our study [21–23].

### Measurement of Lp(a) levels

After blood collection, the samples were immediately sent to our central laboratory and centrifuged, and the Lp(a) concentration (mg/dL) was analyzed in serum. For the remainder of the manuscript, Lp(a) is used instead of Lp(a) levels. Lp(a) was quantified using a particle-enhanced immunonephelometric method using the BN II system from Siemens Healthcare (Eschborn, Germany).

### Assessment of coronary artery calcification

As described previously, baseline CAC was assessed by a nonenhanced electron-beam scan (C-100 or C-150 scanner; GE Imatron, San Francisco, CA, USA) [19]. Furthermore, prospective ECG triggering was done at 80% of the RR interval, and at an image acquisition time of 100 ms, contiguous 3 mm thick slices from the pulmonary bifurcation to the apex of the heart were obtained in both scans [24, 25]. Quantification of CAC score was done using the method suggested by Agatston et al. [26]. The analyses were performed using a Virtuoso workstation (Siemens Medical Solutions, Forchheim, Germany). We further addressed the marked right-skewed distribution of CAC by using the log_e_ transformation of CAC score plus 1, as previously suggested [27–30].

### Assessment of risk factors

The risk factors were recorded at baseline. Smoking behavior (smokers (defined as current or past smokers) and nonsmoker) was assessed in detail [24]. Body mass index (BMI) was calculated as weight divided by height square (kg/m^2^). Current and regular use of medication i.e., antihypertensive or lipid-lowering medication, was recorded in a standardized assessment of medication. The resting blood pressure was measured thrice, with the participants seated by using an automated oscillometric blood pressure device (Omron, HEM-705CP-E). The mean of the second and third values was calculated and used in this study [31]. Standardized enzymatic methods were used to determine serum low-density lipoprotein (LDL) cholesterol, high-density lipoprotein (HDL) cholesterol and triglyceride values (ADVIA 1650, Siemens Medical Solutions, Erlangen, Germany). Diabetes was defined as meeting any of following 4 criteria: (1) participants reported a history of clinically diagnosed diabetes, (2) participants took glucose-lowering drugs, (3) participants had fasting glucose levels of greater than 125 mg/dL, or (4) participants had nonfasting glucose levels of 200 mg/dL or greater [30].

### Genotyping

The participants (*n* = 4331) were genotyped using Illumina GWAS chips (Omni1, OmniExpress, OmniExpress1, HumanCoreExome v1.0 and v1.1) [25, 32]. The 1000 Genomes Project (release October 2014) was used as the reference panel to impute the genetic variations in the study population. Imputation was performed using IMPUTE v2.3.1 software. Thereafter, the data in the PLINK ped format were obtained by specifying the threshold ≥0.9 using GTOOL v0.7.5 [25]. Two *LPA* SNPs (rs10455872: *n* = 3311 and rs3798220: *n* = 3780) and one *IL1F9* SNP (rs13415097: *n* = 3773) were selected from the imputed data. Of the two published *IL1F9* SNPs, only rs13415097 was included in our study, as this SNP was in high LD with rs17659543 (D` = 1 and r^2^ = 1 from our study) [15]. For our analyses, we included 3799 participants having information on sex, age and Lp(a). Participants with any missing data were excluded from the respective analysis.

### Statistical analysis

The association between the log-transformed Lp(a) and CAC was assessed using multivariable linear regression. Lp(a) was further categorized into groups using the 90th percentile cut-off (participants with very elevated levels of Lp(a)) of 54.3 mg/dL from our study, i.e., Lp(a) < 54.3 mg/dL and Lp(a) ≥ 54.3 mg/dL. This cut-off was selected as no clear clinical threshold values for Lp(a) have been suggested [18]. The genotype distribution of all the three SNPs was tested for deviations from Hardy-Weinberg equilibrium (HWE) (exact 2-sided *p* > 0.05), and the SNPs were in HWE (rs10455872: *p* = 0.53, rs3798220: *p* = 1 and rs13415097: *p* = 0.65). The minor allele frequency (MAF) in the study participants was 5.4% for rs10455872 (G), 1.5% for rs3798220 (C) and 16.4% for rs13415097 (C). The association between each SNP with i) log-transformed Lp(a) and ii) CAC was assessed under the additive genetic model using linear regression. The models were first adjusted for age and sex, and the full adjustment consisted of age, sex, diabetes, BMI, systolic blood pressure, diastolic blood pressure, smoking, use of antihypertensive medication and lipid-lowering medication, triglyceride, LDL cholesterol and HDL cholesterol.

To test the hypothesis of a causal association between the *LPA* SNPs and CAC, a Mendelian randomization analysis using *LPA* genotypes as an instrumental variable was performed [33]. In our analysis, genetically determined Lp(a) (as predicted by the *LPA* SNPs) was regressed against the CAC. The inverse-variance weighted (IVW) method was used using the summary statistics (beta coefficients and standard error) for the associations of the two *LPA* SNPs with Lp(a) (exposure) from Clarke et al.’s study [4] and CAC (outcome) from the present study.

We controlled for multiple testing at 5% for our main question regarding the association between the three SNPs and CAC adjusting for age and sex. Consequently, we corrected for three statistical tests that translate into *α*_*BF*_ = 0.0167 using the Bonferroni procedure.

We performed power calculation using QUANTO Version 1.2.4 (http://hydra.usc.edu/gxe) considering a MAF of ≥5% and *α*_*BF*_ = 0.0167 (two-sided). For a sample of 2116 participants (those with CAC > 0), the comparison wise power estimate was 97% (or 67%) assuming a standard normally distributed quantitative trait locus and a standardized effect size of 0.3 (or 0.2) in units of standard deviations (SD) for each risk allele under an additive mode of inheritance without dominance effects. Thus, our study was powered to detect a relatively strong effect size of quantitative CAC predisposing variants when controlling for multiple testing.

Since rs10455872 has been associated with AVC [15], we performed sensitivity analyses by excluding the participants with the presence of AVC (*N* = 464) at baseline in the analyses testing the association between rs10455872 and CAC.

Continuous data are presented as the mean ± SD or median (first quartile: Q1, third quartile: Q3) for skewed data. Accordingly, tests for group differences in the continuous parameters are performed using Student’s *t* test or the Mann-Whitney *U* test. Count data are presented as frequency and percentage, and the group differences are evaluated by using the χ2 or Fisher exact test. Statistical analyses were performed using SAS v.9.4 and PLINK v.19 *(*https://www.cog-genomics.org/plink2*)* [34].

## Results

### Study characteristics

**Fig. 1:**
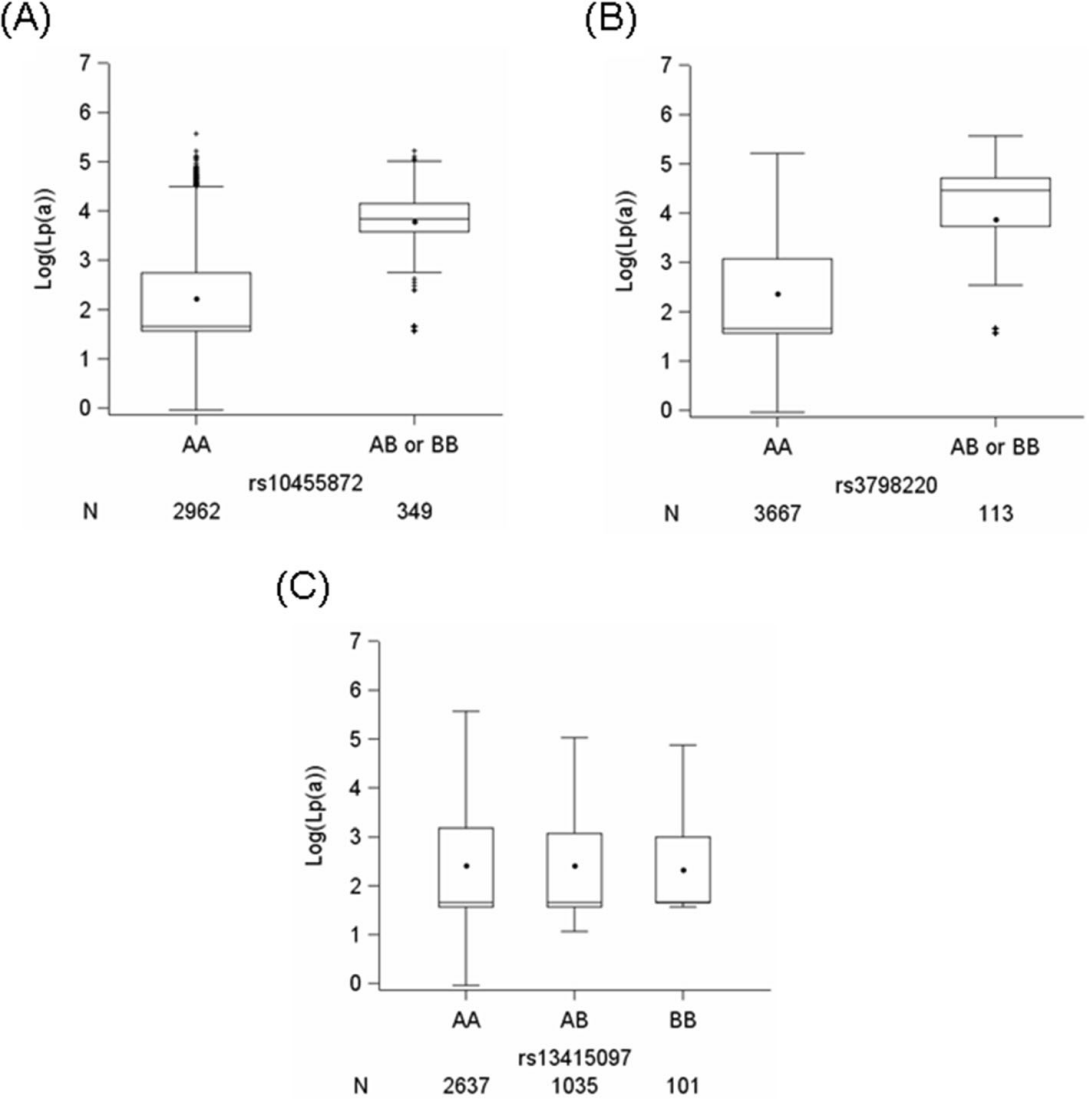
Box plots showing the distribution of log-transformed Lp(a) according to the genotypes. The distribution of log-transformed Lp(a) according to the genotypes (increase in the risk allele) for SNPs (**a**) rs10455872, (**b**) rs3798220 and (**c**) rs13415097. The genotypes are as follows: rs10455872: AA = AA; AB or BB = AG + GG, rs3798220: AA = TT; AB or BB = TC + CC and rs13415097: AA = TT; AB = TC; BB=CC. N denotes the number of participants

The basic characteristics of the Heinz Nixdorf Recall study participants are shown in Table 1. In our study, 3799 and 3639 participants had measurements of Lp(a) and CAC, respectively. Differences in LDL cholesterol, HDL cholesterol, total cholesterol and use of lipid-lowering medication were observed in the Lp(a) stratified groups (Table 1). Figure 1 shows the distribution of log-transformed Lp(a) according to the genotypes for all three SNPs. For rs10455872 and rs3798220, due to the smaller numbers of participants having both risk alleles (BB), we combined the BB genotypes with the heterozygous genotype (AB). With every increase in the risk allele for rs10455872 (median (Q1; Q3): AA: 1.66 (1.57; 2.74) and AB or BB: 3.84 (3.58; 4.15)) and rs3798220 (AA: 1.66 (1.57; 3.09) and AB or BB: 4.46 (3.73; 4.71)), the log-transformed Lp(a) was increased. However, for rs13415097, we did not find any impact of genotypes on the levels of Lp(a). Supplementary Figure 1 A and B additionally show the distribution of Lp(a) by genotype for both *LPA* SNPs. The genotypes for rs10455872 (Supplementary Figure 1A) show better separation of Lp(a) compared to the genotypes for rs3798220 (Supplementary Figure 1B).

**Table 1:** Basic characteristics of the Heinz Nixdorf Recall study participants

	Unstratified (*n* = 3799)	Lp(a) < 54.3 mg/dL (*n* = 3418)	Lp(a) ≥ 54.3 mg/dL (*n* = 381)	*p*^*c*^
Males N (%)	1788 (47.1)	1619 (47.4)	169 (44.4)	0.28
Age (years) ^a^	59.0 ± 7.7	59.0 ± 7.7	59.3 ± 7.8	0.45
BMI (kg/m^2^) ^a^	27.8 ± 4.7	27.8 ± 4.7	27.4 ± 4.5	0.07
Diabetes N (%)	452 (11.9)	408 (11.9)	44 (11.6)	0.87
Antihypertension medication	1159 (30.5)	1035 (30.3)	124 (32.6)	0.38
Diastolic blood pressure (mmHg)^a^	81.5 ± 10.7	81.5 ± 10.7	81.6 ± 11.0	0.70
Systolic blood pressure (mmHg)^a^	132.3 ± 20.5	132.3 ± 20.6	132.6 ± 20.3	0.63
Lipid-lowering medication	334 (9.4)	286 (9.0)	48 (13.4)	0.01
LDL cholesterol (mg/dL)^a^	146.8 ± 36.4	145.8 ± 36.3	156.2 ± 35.8	< 0.001
HDL cholesterol (mg/dL)^a^	59.1 ± 17.3	58.8 ± 17.3	61.4 ± 17.0	0.001
Total cholesterol (mg/dL)^a^	231.5 ± 39.1	230.3 ± 39.0	242.3 ± 38.3	< 0.001
Triglyceride (mg/dL)^b^	123 (88; 177)	123 (89; 176)	124.5 (87.5; 183.0)	0.56
Smoking	2139 (56.4)	1927 (56.4)	212 (55.8)	0.83
Lp(a) (mg/dL)^a^	19.9 ± 26.0	12.7 ± 12.3	84.4 ± 28.2	NA
Lp(a) (mg/dL)^b^	5.3 (4.8; 23.3)	5.3 (4.8; 16.2)	75.2 (62.5; 98.3)	NA
Log (Lp(a))^a^	2.4 ± 1.0	2.2 ± 0.8	4.4 ± 0.3	NA
Log (Lp(a))^b^	1.7 (1.6; 3.1)	1.7 (1.6; 2.8)	4.3 (4.1; 4.6)	NA
CAC (Agatston)^b^	10.8 (0; 107.0)	10.8 (0; 104.9)	11.5 (0; 166.5)	0.17
Log (CAC + 1)^b^	2.5 (0; 4.7)	2.5 (0; 4.7)	2.5 (0; 5.1)	0.17
**rs10455872 (*n*** **= 3311)**				
AA (%)	2962 (90.5)	2775 (92.5)	187 (60.1)	
AB or BB (%)	349 (10.5)	225 (7.5)	124 (39.9)	< 0.0001
**rs3798220 (*n*** **= 3780)**				
AA (%)	3667 (97.01)	3368 (99.0)	299 (79.1)	
AB or BB (%)	113 (2.96)	34 (1.0)	79 (20.6)	< 0.0001
**rs13415097 (*n*** **= 3773)**				
AA (%)	2637 (69.9)	2372 (69.9)	265 (70.1)	
AB (%)	1035 (27.4)	928 (27.3)	107 (28.3)	
BB (%)	101 (2.7)	95 (2.8)	6 (1.6)	0.64

*BMI* Body mass index, *LDL* Low density lipoprotein, *HDL* High density lipoprotein. Data are given as numbers (percentages) unless otherwise indicated. ^a^Data are given as the mean ± SD. ^b^Data are given as the median (Q1; Q3). Lp(a) at the 90th percentile is 54.3 mg/dL. The genotypes are as follows: rs10455872: AA = AA; AB or BB = AG + GG, rs3798220: AA = TT; AB or BB = TC + CC, and rs13415097: AA = TT; AB = TC; BB=CC. ^c^*p* are for differences between Lp(a) stratified groups using χ2 or Fisher exact test, *t* test or Mann-Whitney *U* test. *NA* Not applicable

**Fig. 2:**
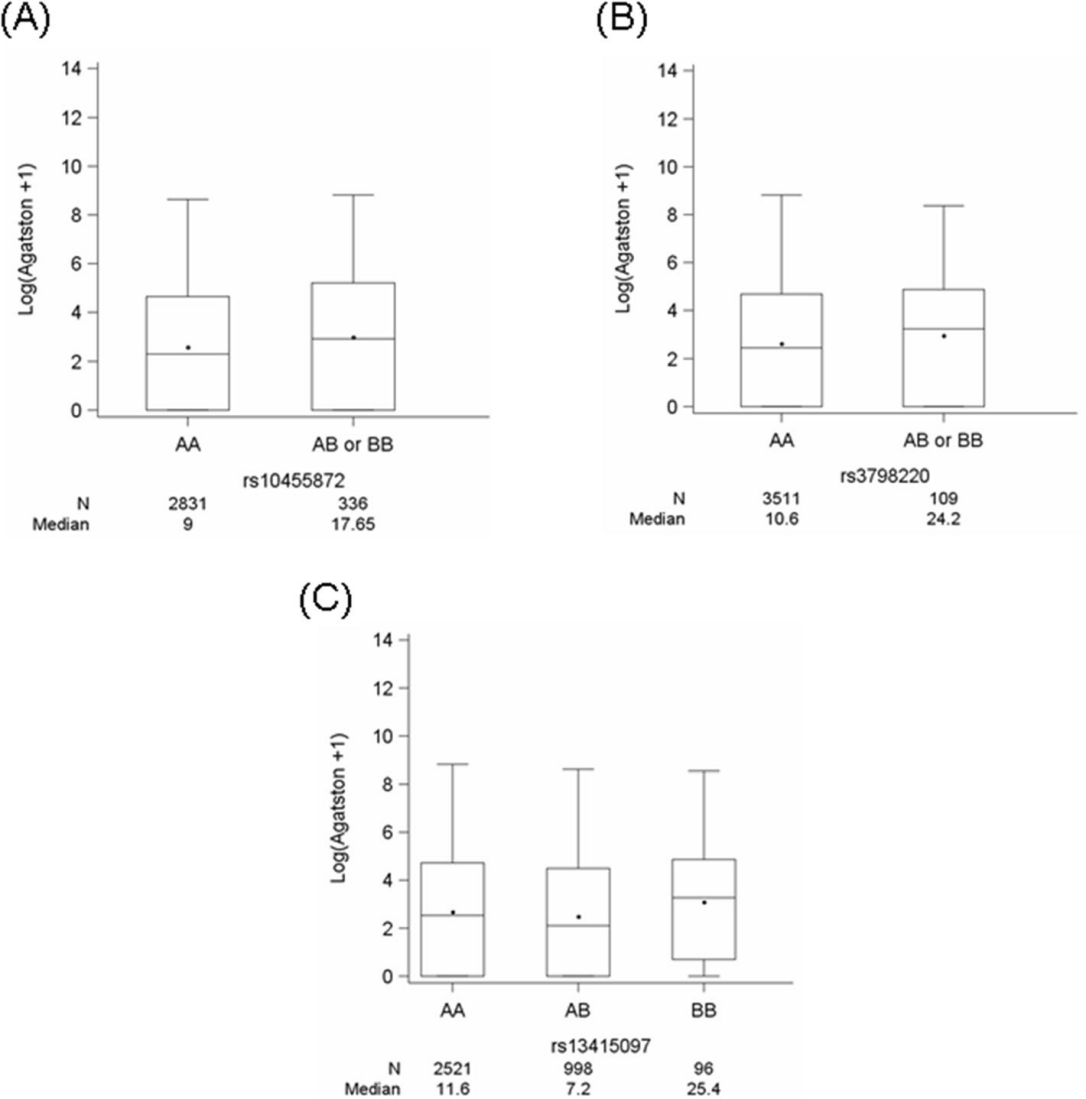
Box plots showing the distribution of CAC (log (CAC + 1)) according to the genotypes. The distribution of CAC (log (CAC + 1)) according to the genotypes (increase in the risk allele) for SNPs (**a**) rs10455872, (**b**) rs3798220 and (**c**) rs13415097. The genotypes are as follows: rs10455872: AA = AA; AB or BB = AG + GG, rs3798220: AA = TT; AB or BB = TC + CC and rs13415097: AA = TT; AB = TC; BB=CC. N denotes the number of participants, and the median is the median value of the CAC score (Agatston units)

Figure 2 shows the distribution of the genotypes for the three SNPs with CAC. For both *LPA* SNPs, with every increase in the risk allele, the CAC score (Agatston) also increased (median (Q1; Q3): rs10455872_AA: 9.0 (0; 102.9) and rs10455872_AB or BB: 17.65 (0; 181.7) and (rs3798220_AA: 10.6 (0; 106.5) and rs3798220_AB or BB: 24.2 (0; 129.7)). However, for rs13415097, we did not find any impact of genotypes on the CAC score. Furthermore, the following observations were made in Lp(a) strata (Supplementary Figure 2A and B). For rs10455872, the Lp(a) < 54.3 mg/dL stratum had a median CAC score (Agatston) of 9.0 (0; 101.3) for AA and 18.1 (0; 165.5) for AB or BB (Supplementary Figure 2A). Similarly, the Lp(a) ≥ 54.3 mg/dL stratum had median a CAC score (Agatston) of 9.3 (0; 126.9) for AA and 13.9 (0; 203.7) for AB or BB (Supplementary Figure 2B). For rs3798220, the Lp(a) < 54.3 mg/dL stratum had a median CAC score (Agatston) of 10.8 (0; 105.2) for AA and 4.6 (1; 66.3) for AB or BB (Supplementary Figure 2A). Similarly, the Lp(a) ≥ 54.3 mg/dL stratum had a median CAC score (Agatston) of 8.8 (0; 169.9) for AA and 39.3 (0; 164.5) for AB or BB (Supplementary Figure 2B).

### Association of genetic variants with coronary artery calcification

In the age- and sex-adjusted analysis, the SNP rs10455872 was statistically significantly associated with CAC (beta per allele = 0.37 [95% CI] [0.14; 0.61], *p* = 0.002) (Table 2). After adjustment for age, sex and Lp(a) levels, the association between the SNP and CAC was attenuated. The association between rs10455872 and CAC showed borderline statistical significance (0.26 [− 0.01; 0.53], *p* = 0.06) (data not shown). SNP rs3798220 did not show any statistically significant association with CAC, although the effect size was high (Table 2). SNP rs13415097 did not show any statistically significant association with CAC (Table 2). The association between rs10455872 and CAC remained significant at a nominal level even after adjusting for risk factors (0.24 [0.01; 0.48], *p* = 0.04) (age, sex, smoking, BMI, HDL cholesterol, LDL cholesterol, triglyceride, diabetes, systolic blood pressure, diastolic blood pressure, antihypertensive medication and lipid-lowering medication) (data not shown).

**Table 2:** Association between *LPA* and *IL1F9* single nucleotide polymorphisms with log_e_ (CAC Score+ 1)

Gene	SNP	N	Unstratified Beta [95% CI], *p*
*LPA*	rs10455872	3167	0.37 [0.14; 0.61], 0.002
*LPA*	rs3798220	3620	0.22 [−0.17; 0.62], 0.26
*IL1F9*	rs13415097	3615	−0.09 [− 0.22; 0.04], 0.19

*SNP* Single nucleotide polymorphism, N: total number of participants in the analysis. The models are adjusted for age and sex

Since rs10455872 is associated with AVC, we performed sensitivity analyses by excluding the participants with the presence of AVC at baseline. In the age- and sex-adjusted analysis, the association of rs10455872 with CAC remained significant at the nominal level (0.30 [0.05; 0.55], *p =* 0.018) (data not shown). Further adjustment for Lp(a) attenuated the association (0.22 [− 0.07; 0.51], *p =* 0.13) (data not shown).

### Association of Lp(a) with coronary artery calcification

Table 3 shows the association of Lp(a) with CAC. Log-transformed Lp(a) (beta per log unit increase in Lp(a) = 0.11 [95% CI] [0.04; 0.18], *p* = 0.002) and categories of Lp(a) (Lp(a) ≥ 54.3 mg/dL vs. Lp(a) < 54.3 mg/dL) (0.23 [0.005; 0.45], *p* = 0.05) were statistically significantly associated with CAC in the age- and sex-adjusted analyses. The additional phenotypic variance explained by the addition of log-transformed Lp(a) or Lp(a) categories to the base model (age and sex: R^2^ = 25.3%) was 0.2 and 0.1%, respectively. Supplementary Figure 3 additionally shows the association between the log-transformed Lp(a) and CAC in an unadjusted linear analysis that resulted in an estimate of 0.09 [0.01; 0.17], *p* = 0.03, similar to the adjusted analysis (Table 3). The association between log-transformed Lp(a) and CAC (0.09 [0.02; 0.16], *p* = 0.008) remained statistically significant even after adjusting for risk factors (age, sex, smoking, BMI, HDL cholesterol, LDL cholesterol, triglyceride, diabetes, systolic blood pressure, diastolic blood pressure, antihypertensive medication and lipid-lowering medication) (data not shown). However, the association between Lp(a) categories and CAC was not statistically significant after adjusting for risk factors (0.18 [− 0.05; 0.40], *p* = 0.13) (data not shown). As a sensitivity analysis, we looked at the association between Lp(a) and CAC in quantiles of CAC in an unadjusted model (Supplementary Figure 4). Within a given CAC quantile, the value of CAC increases with increasing Lp(a).

**Table 3:** Association between Lp(a) and log_e_ (CAC Score+ 1)

	N	Beta [95% CI], *p*	Explained variance (%)
Base	3636		R^2^ = 25.3
Lp(a)^a^	3639	0.11 [0.04; 0.18], 0.002	0.2
Base	3639		R^2^ = 25.3
Lp(a)^b^	3639	0.23 [0.005; 0.45], 0.05	0.1

N: total number of participants. The models are adjusted for age and sex. Base: without Lp(a) adjusted for age and sex. ^a^log-transformed Lp(a) levels, ^b^Lp(a) ≥ 54.3 mg/dL vs. Lp(a) < 54.3 mg/dL. Explained variance is the difference in R^2^ between each of the models and the base model

### Association of genetic variants with Lp(a)

The associations of SNPs with log-transformed Lp(a) are listed in Table 4. *LPA* rs10455872 and rs3798220 in the age- and sex-adjusted analyses were statistically significantly associated with log-transformed Lp(a) (beta per allele [95% CI], *p*: beta_rs10455872_: 1.56 [1.46; 1.65], *p* < 0.0001 and beta_rs3798220_: 1.51 [1.33; 1.69], *p* < 0.0001). The SNP rs10455872 in the age- and sex-adjusted analysis explained 24% of the variance in Lp(a). However, rs3798220 explained only 6.9% of the variance in Lp(a). The *IL1F9* (rs13415097) SNP however, did not show any statistically significant association with Lp(a) (Table 4). The association between rs10455872 and rs3798220 with log-transformed Lp(a) remained statistically significant even after adjusting for risk factors (age, sex, smoking, BMI, HDL cholesterol, LDL cholesterol, triglyceride, diabetes, systolic blood pressure, diastolic blood pressure, antihypertensive medication and lipid-lowering medication) (beta_rs10455872_: 1.53 [1.43; 1.63], *p* < 0.0001 and beta_rs3798220_: 1.46 [1.28; 1.64], *p* < 0.0001) (data not shown).

**Table 4:** Association between *LPA* and *IL1F9* single nucleotide polymorphisms with log-transformed Lp(a)

SNP	N	Beta [95% CI], *p*	Explained variance (%)
rs10455872	3311	1.56 [1.46; 1.65], < 0.0001	24
rs3798220	3780	1.51 [1.33; 1.69], < 0.0001	6.9
rs13415097	3773	−0.01 [−0.08; 0.05], 0.63	0.15

*SNP* Single nucleotide polymorphism, N: total number of participants in the analysis. The models are adjusted for age and sex

### Mendelian randomization using genetically determined Lp(a) with coronary artery calcification

The Mendelian randomization analysis using the IVW method showed that Lp(a) is a causal risk factor for CAC, with an estimate of 0.27 per log-unit increase in Lp(a) levels (estimate (95% CI), *p*: 0.27 [0.11; 0.44], *p* = 0.001) (Table 5).

**Table 5:** Causal estimates of Lp(a) on CAC from Mendelian randomization analysis

	CAC		
	Causal estimate	95% CI	*p*
IVW	0.27	[0.11; 0.44]	0.001

*CAC* Coronary artery calcification, *IVW* Inverse-variance weighted. Lp(a) was log-transformed

## Discussion

In a large population-based Heinz Nixdorf Recall study, we investigated the association of Lp(a), *LPA* (rs10455872 and rs3798220) and *IL1F9* (rs13415097) SNPs with coronary artery calcification. In our study, we found that i) *LPA* rs10455872 is associated with CAC, ii) the association between rs10455872 and CAC was attenuated after adjustment for Lp(a), iii) Lp(a) also showed an association with CAC, iv) both *LPA* SNPs were associated with Lp(a) and v) we did not find any evidence of an association of *IL1F9* rs13415097 with Lp(a) or CAC. The association between rs10455872 and CAC remained statistically significant even after controlling for multiple testing. Using a Mendelian randomization approach, we found that genetically determined Lp(a) levels were causally associated with CAC.

Lp(a) is a cholesterol-rich particle having a covalently linked molecule of apolipoprotein B100 with a molecule of apo(a). We confirmed the previous association of both the *LPA* SNPs with the levels of Lp(a). Similar to a previous study, rs10455872 explained approximately 24% and rs3798220 explained 6.4% of the total variance of Lp(a) [4]. Observational studies have shown the association of Lp(a) with the risk of CAD [2, 3, 35, 36]. Moreover, genetic studies have shown the association of genetic variants in *LPA* with a higher risk for CAD, providing evidence for a causal role of Lp(a) in CAD [4–6]. Additionally, several observational studies looked at the role of Lp(a) on CAC, an important predictor for CAD. The result of the association between Lp(a) and CAC from the present study fits the findings of the studies showing a positive association between Lp(a) and CAC [10–12, 37, 38]. However, none of the observational studies systematically examined the association between Lp(a) and *LPA* genetic variants with CAC. Our study is the first to examine the association of Lp(a) as well as *LPA* genetic variants with CAC. Of the two *LPA* SNPs, only rs10455872 showed a statistically significant association with CAC. The association between rs10455872 and CAC was attenuated after adjusting for Lp(a), showing that Lp(a) levels mediate the effect of the rs10455872 SNP on CAC. The genetic association of the *LPA* variant with CAC provides evidence from a previous study showing that patients with CAD carrying *LPA* risk alleles have increased susceptibility to atherosclerotic manifestations and are more likely to be diagnosed earlier with CAD than are CAD cases not carrying these variants [39]. In addition, in vivo and in vitro studies have provided evidence that Lp(a) is present in coronary atherosclerotic plaques and plays a role in plaque inflammation and instability in atherosclerotic coronary arteries [40, 41]. The data of our study suggest that lifelong elevated levels of Lp(a) due to the *LPA* rs10455872 SNP might lead to an increase in coronary artery calcification that further leads to CVD events. However, it will be interesting to see if the results of our study could be replicated in other larger samples.

The present study is a population-based cohort study with data on Lp(a) levels, *LPA* and *IL1F9* SNPs and measurement of CAC. Given the different distributions of CAC in men and women gender-specific effects can be detected for CAC; however, due to moderate sample size gender stratified analyses could not be carried out in this study [11, 42].

## Conclusions

In conclusion, we provide evidence for the association of *LPA* rs10455872, which is strongly associated with higher Lp(a) levels, and CAD is associated with higher levels of Lp(a) and CAC in our study. Our findings show that the rs10455872 SNP, through elevated Lp(a) levels, might play a role in promoting the development of atherosclerosis leading to CVD events.

## Supplementary information

**Additional file 1: Figure S1.** Distribution of Lp(a) (mg/dL) according to the genotypes for *LPA* rs10455872 and rs3798220. **Figure S2.** Distribution of CAC (log(CAC + 1)) in strata of Lp(a) according to the genotypes for SNPs rs10455872, rs3798220 and rs13415097. **Figure S3.** Association between log-transformed Lp(a) with log(CAC + 1) in an unadjusted model. **Figure S4.** Association between Lp(a) and CAC score (Agatston) in quantiles of CAC in an unadjusted model.

## Data Availability

Due to data security reasons, i.e., the data contain potentially participant identifying information; the Heinz Nixdorf Recall study does not allow sharing data as a public use file. However, other authors/researchers are allowed to access data upon request, which is the same way the authors of the present paper obtained the data. Data requests can be addressed to recall@uk-essen.de.

## Abbreviations

Lp(a): Lipoprotein(a)
*LPA*: Lipoprotein (a) gene
*IL1F9*: Interleukin 1 family, member 9
CAC: Coronary artery calcification
SNP: Single nucleotide polymorphism
apo(a): apolipoprotein
CAD: Coronary artery disease
GWAS: Genome-wide association studies
CVD: Cardiovascular disease
AVC: Aortic value calcification
MVC: Mitral annular calcification
CT: Computer tomography
BMI: Body mass index
LDL: Low density lipoprotein
HDL: High density lipoprotein
IVW: Inverse-variance weighted
HWE: HARDY-Weinberg equilibrium
MAF: Minor allele frequency
