# Supplementary material for: Association between lipoprotein(a) (Lp(a)) levels and Lp(a) genetic variants with coronary artery calcification
Source: BMC Med Genet. 2020 Mar 27;21:62. doi: 10.1186/s12881-020-01003-3 (PMC7099786; doi:10.1186/s12881-020-01003-3)

Additional file

**Supplementary Figures 1: Distribution of Lp(a) (mg/dL) according to the genotypes for *LPA* rs10455872 and rs3798220**


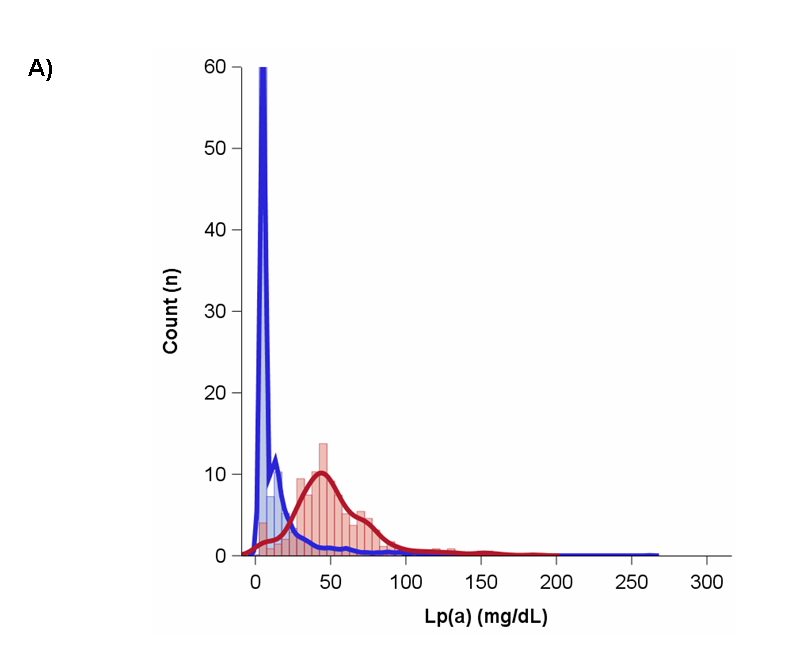


**(A)**

**rs10455872**


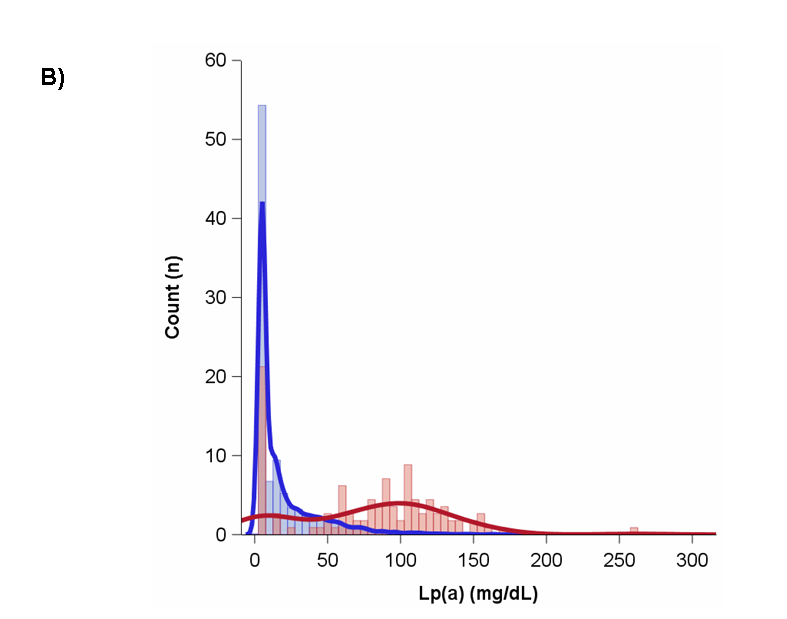


**(B)**

**rs3798220**

Supplementary Figures 1 (A) and (B): Distribution of Lp(a) (mg/dL) according to the genotypes for *LPA* (A) rs10455872 and (B) rs3798220. The blue and red curves are AA and AB or BB genotypes respectively. The genotypes are as follows rs10455872: AA=AA; AB or BB=AG+GG and rs3798220: AA=TT; AB or BB=TC+CC.

**Supplementary Figures 2: Distribution of CAC (log(CAC+1)) in strata of Lp(a) according to the genotypes for SNPs rs10455872, rs3798220 and rs13415097**

**(A)**
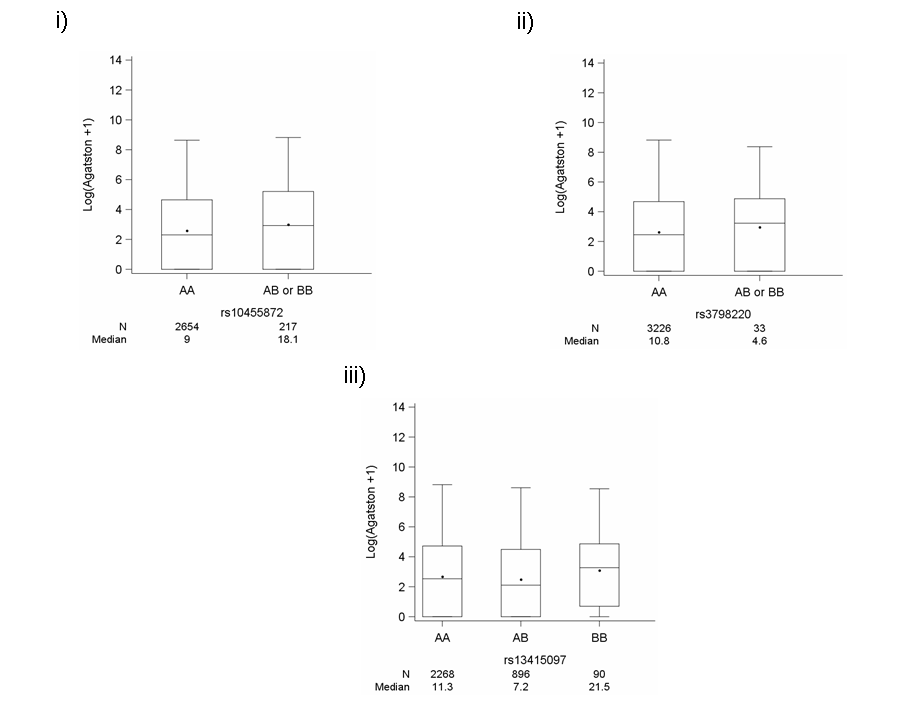


**(B)**
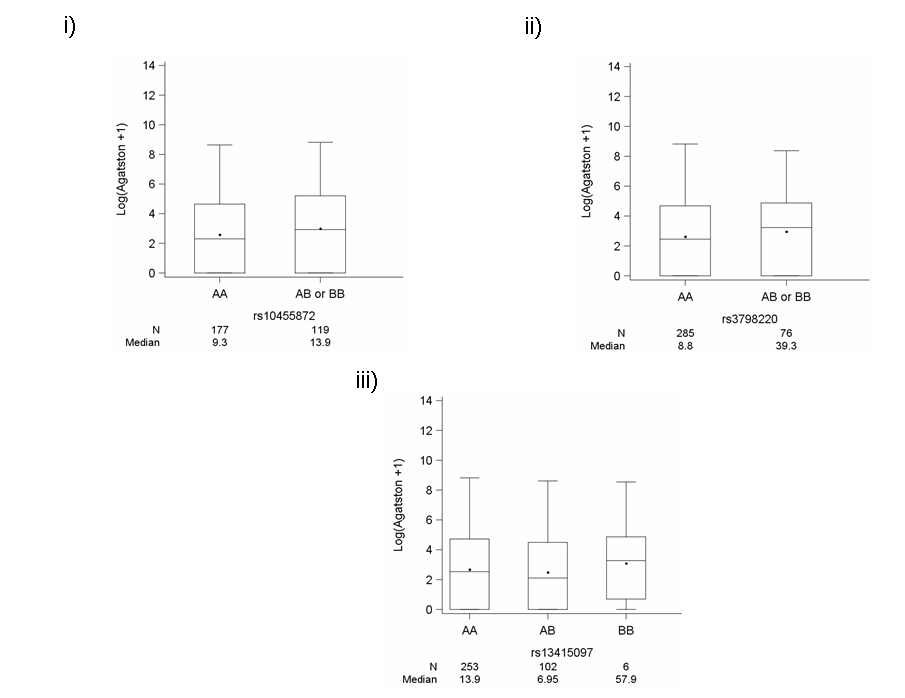


Supplementary Figures 2 (A) and (B): Distribution of CAC (log(CAC+1)) in strata of Lp(a); (A) Lp(a)<54.3 mg/dL and (B) Lp(a) ≥54.3 mg/dL; according to the genotypes (increase in the risk allele) for SNPs i) rs10455872, ii) rs3798220 and iii) rs13415097. The genotypes are as follows rs10455872: AA=AA; AB or BB=AG+GG, rs3798220: AA=TT; AB or BB=TC+CC and rs13415097: AA=TT; AB=TC; BB=CC. N denotes the number of participants and median is the median value of CAC score (Agatston units).

**Supplementary Figure 3: Association between log-transformed Lp(a) with log(CAC+1) in an unadjusted model.**


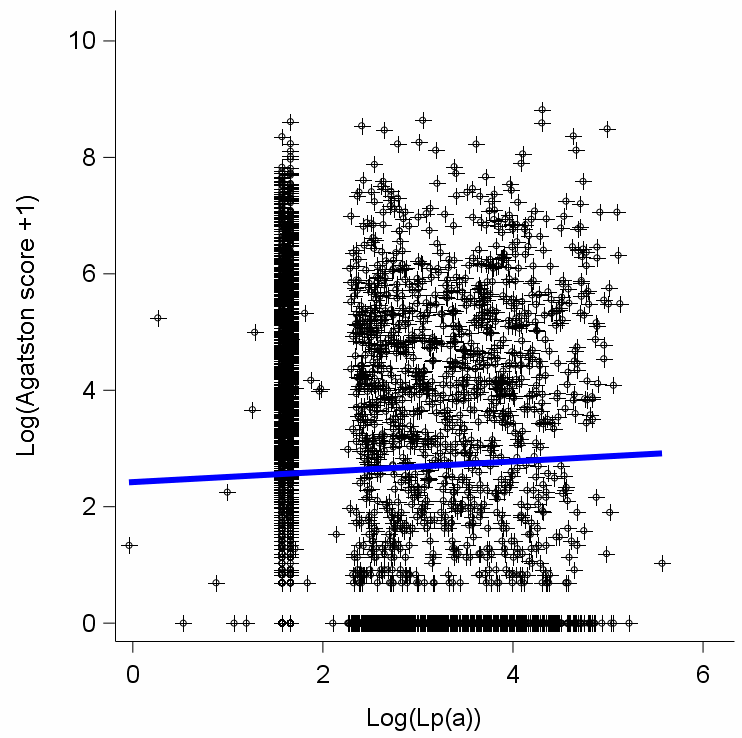


Log(Agatston score+1)= 2.42 +

0.09 * Log(Lp(a))

**Supplementary Figure 4: Association between Lp(a) and CAC score (Agatston) in quantiles of CAC in an unadjusted model.**


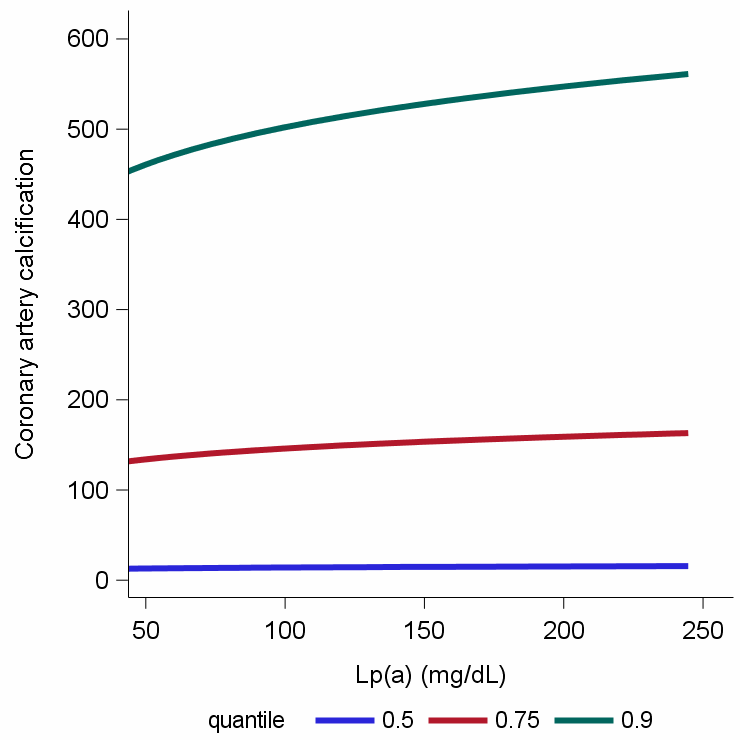

Supplement: Supplementary file 1 — Additional file 1: Figure S1. Distribution of Lp(a) (mg/dL) according to the genotypes for LPA rs10455872 and rs3798220. Figure S2. Distribution of CAC (log(CAC + 1)) in strata of Lp(a) according to the genotypes for SNPs rs10455872, rs3798220 and rs13415097. Figure S3. Association between log-transformed Lp(a) with log(CAC + 1) in an unadjusted model. Figure S4. Association between Lp(a) and CAC score (Agatston) in quantiles of CAC in an unadjusted model. [file 12881_2020_1003_MOESM1_ESM.docx]
